# High-frequency oscillations in epileptic and non-epileptic Alzheimer's disease patients and the differential effect of levetiracetam on the oscillations

**DOI:** 10.1093/braincomms/fcaf041

**Published:** 2025-02-13

**Authors:** M C Vishnu Shandilya, Kwaku Addo-Osafo, Kamalini G Ranasinghe, Mohamad Shamas, Richard Staba, Srikantan S Nagarajan, Keith Vossel

**Affiliations:** Department of Neurology, David Geffen School of Medicine, University of California, Los Angeles 90095, USA; Department of Neurology, David Geffen School of Medicine, University of California, Los Angeles 90095, USA; Department of Radiology and Biomedical Imaging, University of California, San Francisco 94143, USA; Department of Neurology, David Geffen School of Medicine, University of California, Los Angeles 90095, USA; Department of Neurology, David Geffen School of Medicine, University of California, Los Angeles 90095, USA; Department of Radiology and Biomedical Imaging, University of California, San Francisco 94143, USA; Department of Neurology, David Geffen School of Medicine, University of California, Los Angeles 90095, USA

**Keywords:** high-frequency oscillations, Alzheimer’s disease, epilepsy, levetiracetam, hyperactivity

## Abstract

Alzheimer's disease increases the risk of developing epilepsy together with cognitive decline. Early diagnosis or prediction of parameters associated with epileptic activity can greatly help in managing disease outcomes. Network hyperexcitability is a candidate of interest as a neurophysiological biomarker of Alzheimer's disease. High-frequency oscillations are increasingly recognized as potential biomarkers of hyperexcitability and epileptic activity. However, they have not yet been identified in Alzheimer's disease. In this study, we measured high-frequency oscillations via magnetoencephalography recordings in Alzheimer's disease patients with and without epileptic activity, as part of a Phase 2a randomized, double blind clinical trial of the efficacy of levetiracetam to improve cognitive functions in Alzheimer's disease. To measure the high-frequency oscillations, we used 10-min magnetoencephalography recordings (275-channel and sampling rate 1200–4000 Hz) during awake resting periods in participants with Alzheimer's disease and healthy controls. Recordings from 14 Alzheimer's disease participants, with six having non-epileptic Alzheimer's disease (median age: 60.8, 2 M/4 F), eight having sub-clinical epileptic activity (median age: 54.9, 5 M/3 F) and eight as control (median age: 71, 5 M/3 F), were analysed using two software scripts: Delphos and a custom-made script, for detecting high-frequency oscillations. Levetiracetam 125 mg twice-a-day or placebo was administered for 4 weeks in between two magnetoencephalography recordings, and 4 weeks of washout before switching levetiracetam/placebo phases for each participant. High-frequency oscillations were categorized into ripples (80 to 250 Hz) and fast ripples (250 to 500 Hz). At baseline, Alzheimer's disease participants, both epileptic and non-epileptic had higher rate of ripples and fast ripples than controls in several left/right hemispheric sensor regions (*P* < 0.05). Additionally, compared to epileptic, non-epileptic had higher rate of ripples in left-frontal, left-temporal and cerebral fissure regions and higher rate of fast ripples in left-frontal regions (*P* < 0.05). In epileptic type, levetiracetam decreased ripples in bilateral-frontal, bilateral-occipital regions and cerebral fissure, whereas in non-epileptic type, levetiracetam increased both ripples and fast ripples in right central and left parietal regions, and ripples in the right parietal region (*P* < 0.05). Additionally, we found hemisphere asymmetry in epileptic type, with right temporal/occipital having more high-frequency oscillations than their counterpart region. Overall, Alzheimer's disease had a high level of high-frequency oscillations, with higher numbers observed in non-epileptic type. Levetiracetam decreased high-frequency oscillations in epileptic but increased high-frequency oscillations in non-epileptic. Thus, high-frequency oscillations can function as a biomarker of hyperexcitability in Alzheimer's disease and may be more pathological when asymmetric and coinciding with presence of epileptic activity. Levetiracetam has the potential for treating hyperactivity in patients with epileptic Alzheimer's disease.

## Teaser

Shandilya MCV *et al*. report that Alzheimer's disease exhibits increased high-frequency oscillations in both non-epileptic and epileptic cases. Levetiracetam reduces these oscillations in epileptic cases but raises them in non-epileptic cases. They suggest high-frequency oscillations as a biomarker for Alzheimer’s, useful for studying molecular and neurophysiological variables associated with hyperexcitability.

## Graphical Abstract

**graphical abstract:**
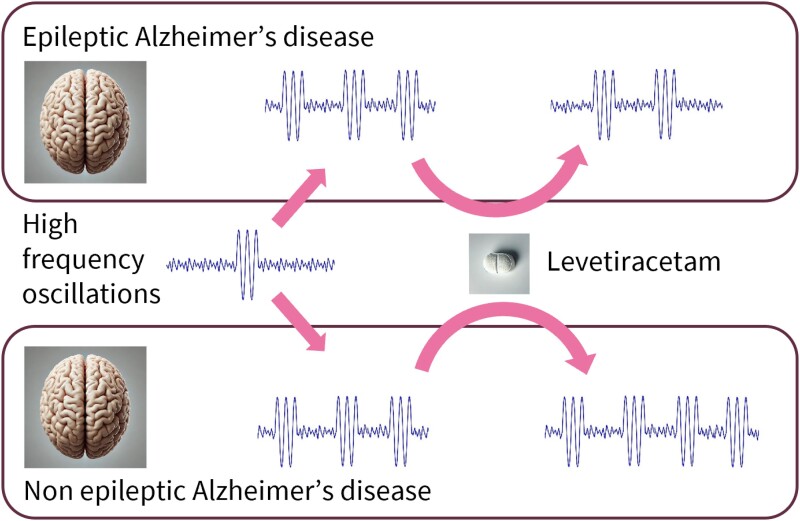

## Introduction

Having Alzheimer's disease increases the risk of developing epilepsy by around 3-fold,^1^ with a significant proportion (23–62%) experiencing seizures in parallel with cognitive decline.^2,3^ Seizures also accelerate cognitive decline in Alzheimer's disease, and early diagnosis or prediction of parameters associated with epileptic activity can greatly help in managing disease outcomes.^4,5^ Anti-seizure medications can improve cognitive functions and may slow cognitive decline in patients with Alzheimer's disease and epileptic activity.^4,6-8^ Seizures and sub-clinical epileptic activity are a manifestation of neuronal network hyperexcitability,^6,9^ a state characterized by an imbalance between excitatory and inhibitory processes in the brain. Neuronal hyperexcitability can spread Alzheimer's disease pathology,^10^ and an early correction to reduce this activity correlates with reduced memory loss in mouse models of Alzheimer's disease.^11^ Aberrant neuronal activity is also associated with early stages of Alzheimer's disease,^12^ making it a candidate of interest as a neurophysiological biomarker.

There is a need for new biomarkers of hyperexcitability in Alzheimer's disease, as it is a treatable factor in cognitive decline associated with the disease. High-frequency oscillations (HFOs) are increasingly recognized as potential biomarkers of hyperexcitability and epileptic activity.^13-15^ HFOs are observed both within and outside clinically defined seizure onset zones, with varying degrees of association to the epileptogenic zone (EZ),^16,17^ in both animal models and patients with refractory focal epilepsy^18,19^; however, HFOs, particularly fast ripples (FR), in the EZ have more spectral power than those outside the zone,^20^ and they are used to localize the EZ to improve surgical success in drug-resistant epilepsy patients.^21^ HFOs were originally discovered using microelectrode array recordings,^22-25^ and currently they are categorized by frequency into ripples (80–250 Hz) and FR (250–500 Hz).^26-29^ HFOs are spontaneous, rapid and transient brain waves, identifiable from the background signal, with clear patterns observed using intracranial recordings.^15,16,30,31^ Ripples (R) are associated with memory consolidation off-task,^32,33^ memory retrieval on-task,^34,35^ and represent inhibitory post-synaptic potentials.^36,37^ They can be both physiological and pathophysiological. When observed in intracranial EEG recordings, epileptic activity is more associated with R that occur on a flat background than those occurring on a non-flat background.^38^ In contrast, FR are generally considered pathophysiological and associated with epileptic activity and atrophy,^26,36,39-41^ but their presence is observed in non-epileptic regions of epileptic patients.^42^ HFOs have been observed in both cortical and hippocampal regions, with potentially distinct functional roles. While hippocampal ripples are associated with memory consolidation, cortical ripples may be involved in local information processing and cross-regional communication.^34,43^ However, HFOs are yet to be identified in Alzheimer's disease. Scalp EEG and MEG recordings of HFOs have been reported in epilepsy including simultaneous recordings,^44^ but have not been tested in Alzheimer's disease. If they are affected in Alzheimer's disease, they could become a potential neurophysiological biomarker of hyperexcitability in the disease. In this study, we examined HFOs using MEG in Alzheimer's disease patients with and without sub-clinical epileptic activity, which is a part of a Phase 2a randomized, double blind clinical trial of the efficacy of levetiracetam (LEV) to improve cognitive functions in Alzheimer's disease, in which HFOs were not examined. LEV was shown to improve the spatial memory and executive functions in patients with Alzheimer's disease and detectible epileptiform activity (EAD).^4^

## Materials and methods

### Study design and participants

The MEG recordings of Alzheimer's disease participants were derived from the previously published Vossel *et al*.^4^ study of LEV for Alzheimer's disease–associated network hyperexcitability. Briefly, it was a Phase 2a randomized double-blinded placebo-controlled crossover clinical trial conducted at the University of California, San Francisco (UCSF) and the University of Minnesota. It followed the Consolidated Standards of Reporting Trials (CONSORT) reporting guidelines for randomized clinical trials and was conducted in accordance with the Declaration of Helsinki.^45^ Only data from UCSF were included for consistency in magnetoencephalography (MEG) recordings. Alzheimer's disease participants visited UCSF from October 16, 2014, to June 9, 2017. The inclusion criteria include age less than 81; a diagnosis of probable Alzheimer's disease; a Mini-Mental State Examination (MMSE) score of 18 points or higher, and/or a Clinical Dementia Rating (CDR) score of <2 points. The exclusion criteria include participants receiving anti-seizure medications; significant systemic medical illness; and any condition, along with Alzheimer's disease, that could account for cognitive deficits. The MEG recordings of control participants were derived from the Vossel *et al*.^46^ prospective observation clinical study. Controls were recruited between August 2008 and February 2015. The inclusion criteria include MMSE score of ≥28; a CDR-Sum of Boxes score of 0; no reports of cognitive deficits; and no atrophy less than that of age-appropriate level. Participants were grouped as Alzheimer's disease, Alzheimer's disease with EAD (spikes or sharp waves), Alzheimer's disease without epileptiform activity (NEAD), based on results from overnight EEG and 1-hour resting M/EEG exams or history of seizure (one Alzheimer's disease participant), and healthy controls. None of the controls had EAD on their overnight EEG or 1-hour resting M/EEG recordings. The data were analysed at the University of California, Los Angeles (UCLA) after August 2022.

### M/EEG imaging

**Figure 1:**
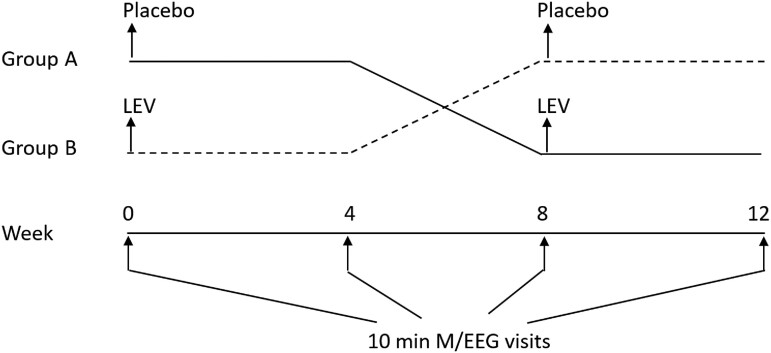
**Switch-over treatment process.** Alzheimer's disease participants had four 10-min MEG scans, with 4-week intervals between each scan. Controls had only one scan. Alzheimer's disease participants received either LEV 125 mg or placebo twice-a-day during the first 4 weeks and no treatment for the next 4 weeks. Then they received the opposite treatment (placebo or LEV) in the final 4 weeks.

Participants underwent 10-min MEG recordings with simultaneous EEG (M/EEG, 275-channel) at high sampling rate (sampling rate 1200–4000 Hz) starting at 40 min into their 60-min resting-state, eyes-closed M/EEG exams. The recorded data are converted from CTF file system to EDF format (using *FieldTrip* MATLAB scripts) for visual inspection and for data processing in MATLAB. This generated 10 1-min EDF segments for MEG (275-channel) and EEG (21-channel) each, filed separately. Alzheimer's disease participants underwent four visits of M/EEG with 4 weeks of interval between each visit (Fig. 1). Controls visited only once.

### Levetiracetam

Alzheimer's disease participants were randomly assigned to the switch-over treatment process (Fig. 1) where they initially received either LEV 125 mg twice-a-day or placebo for 4 weeks, followed by 4 weeks of washout (break period) and then received the opposite treatment for 4 weeks, or the reverse sequence. They had two M/EEG visits for each treatment stage, with 4-week intervals between them. Hence, total M/EEG visits are four. Controls were not administered LEV, and they only visited once for their M/EEG.

### Data analysis

Among 17 Alzheimer's disease participants, we excluded their data from the analysis if they did not undergo all the four visits (n = 2); their M/EEG sampling rate was <1000 Hz—which is required to detect HFOs (n = 2); or if the recorded data was corrupted (n = 1). Among 20 controls, 10 were randomly selected to match for sampling rate of M/EEG. Among these, we excluded the data from the analysis for those whose blood and cerebrospinal fluid sample was unavailable (n = 2).

Initially, the EDF files were visually examined to check the presence of excessive noise, an abnormally high voltage that is present across multiple channels within a small period. As most of the files (∼95%) do not have very high noise, no files were excluded at this stage. Then, the EDF files were processed by *Delphos detector* script,^47^ which detects the HFOs by measuring the peaks in a signal and analyses them by the time width and frequency of the peaks above a threshold score that correspond to a normalized energy value. It begins by applying a Tukey window to reduce spectral leakage, and then calculates a time-frequency (TF) spectrogram. The algorithm removes outliers using interquartile range analysis and normalizes the data by fitting it to a normal distribution. It then employs image processing techniques to detect local maxima in the TF data by comparing each point in the TF data to its eight adjacent neighbours (i.e. time and frequency dimensions). Finally, it classifies these events as ripples or FR based on whether their local maxima fall within the pre-defined frequency bands for each type of oscillation (80–250 Hz for ripples, 250–500 Hz for FR). We set the threshold score to 20. Other standard settings used by the script are: minimum duration for HFO detection (1.4 times the expected oscillation period), maximum frequency spread for HFO (10 frequency divisions), frequency detail level in analysis (12 sub-divisions per doubling of frequency), TF resolution balance (set to 20, higher values favour frequency precision), analysed frequency range (3 octaves, covering 80–500 Hz) and threshold for identifying unusual signal patterns (0.005 for MEG data, where lower values are more selective). *Delphos* has high sensitivity and precision to detect HFOs even in low signal-to-noise ratio,^48^ such as a MEG signal. The output gave a list of potential HFOs (candidates), which were then randomly verified visually. The output files that were >400 kB (around 5% of total files), representing excessive noise, were excluded from further analysis (Supplementary Fig. S1). For the remaining files, we created an additional script in MATLAB to filter out noise by the following criteria: maximum five candidates across all channels within 400 ms, and the candidate with highest power among them was selected; the signal was high-pass filtered at 80 Hz and the standard deviation (SD) was calculated for signal's amplitude for each 400-ms time window. Most of the signals (∼72%) were 1200 Hz; however, those that were higher were downsampled to 1200 Hz. The ratio of the candidate's maximum amplitude to the SD of its respective time window was calculated; candidates having ratios <3 were omitted as they had low amplitude of high-frequency component. HFO rates (averaged over 10-min recording) were analysed group-wise.

### Sleep analysis

For determining awake or sleep from EEG, we filtered the signal using bandpass (1 to 40 Hz) filter and analysed the frequency of eye blinks as: awake if blinks were frequent, drowsy if blinks were occasional and their duration was >0.5 s, and stage-2 or stage-3 sleep if blinks were absent and the tracings had occasional, large amplitude, low frequency and synchronized waves.

### Asymmetry index

We calculated Asymmetry index (AI) for EAD and NEAD to measure the degree of asymmetry of R and FR rates between hemispheres of brain. AI for was calculated using the formula: $AI=200*\;\frac{\left(\;\mathrm{HFO}\;\mathrm{rate}\;\;\mathrm{left}\;\mathrm{hemisphere}\;\text{–}\;\mathrm{HFO}\;\mathrm{rate}\;\;\mathrm{right}\;\mathrm{hemisphere}\right)}{\left(\;\mathrm{HFO}\;\mathrm{rate}\;\mathrm{left}\;\mathrm{hemisphere}+\mathrm{HFO}\;\mathrm{rate}\;\mathrm{right}\;\mathrm{hemisphere}\;\right)}\;$ We compared AI of EAD against NEAD to test the effect of epileptic factor and compared AI of both EAD and NEAD against zero for their respective group effect.

### Statistical analysis

Fisher's test was used to analyse categorical variables. For MEG, the rate of R or FR, were grouped according to their respective lobar region. We calculated the average rate of R or FR of all participants within a cohort for each channel and compared regional channels between cohorts. For analysing absolute AI, we compared a group against a value of zero, using 2-tailed 1-sample Student's *t*-test. We checked normality of data using D'Agostino and Pearson test. For normally distributed data, for comparisons between two groups, we used 2-tailed unpaired Student's *t*-test (ST). For comparisons between three groups, we used ANOVA with Tukey's multiple comparisons test (AT). For data that was not normally distributed, we used Mann–Whitney test (MW) for two groups and Kruskal–Wallis test with Dunn's multiple comparisons test (KD) for three groups. We performed Spearman correlation analyses to assess the similarity across brain regions. For LEV analyzation, we compared drug versus placebo for each group. Analysis was performed using GraphPad Prism v10.2.

## Results

### Data demographics

The demographics of participants analysed are given in Table 1. There were 14 participants with Alzheimer's disease (eight EAD and six NEAD) and eight controls. In Alzheimer's disease, there were equal males (M) and females (F), with seven each. In controls, there were five M and three F. Sex was matched between Alzheimer's disease and controls (*P* = 0.67, Fisher's exact test). Sex was also matched between EAD, NEAD and controls (*P* = 0.64, Fisher's exact test). Controls (mean: 71.7 years) were 12.4 years older than Alzheimer's disease participants (mean: 59.3 years) (*P* < 0.001, unpaired *t*-test). Controls were 12.7 and 11.9 years older than EAD (mean: 58.9 years, *P* = 0.001) and NEAD (mean: 59.8 years, *P* = 0.004), respectively (ANOVA with Tukey's multiple comparisons test). Age was matched between EAD and NEAD (*P* = 0.96, ANOVA, Tukey's). Years of education were matched between Alzheimer's disease (mean: 16.79) and controls (mean: 17.0) (*P* = 0.83, unpaired *t*-test). Years of education were also matched between controls, EAD and NEAD (all *P* > 0.96, ANOVA, Tukey's). Self-identified race and ethnicity of all the participants was white and non-Hispanic/Latinx, respectively. There was no significant difference between sampling rates of MEG recordings between Alzheimer's disease and control (*P* = 0.12) and between EAD and NEAD (*P* = 0.17, unpaired *t*-tests). There were no significant differences between control versus Alzheimer's disease and EAD versus NEAD for handedness (*P* > 0.99) or memantine use (*P* > 0.99) (Fisher's exact test). All Alzheimer's disease participants used acetylcholinesterase inhibitors. EAD (mean: 25.25, *P* = 0.022) and NEAD (mean: 24.33, *P* = 0.009) had lower MMSE scores than controls (mean: 29.25), but no difference between them (*P* = 0.81, ANOVA, Tukey's). No differences were found between EAD and NEAD for CDR (*P* = 0.81) or CDR-Sum of Boxes (*P* = 0.38) (unpaired *t*-tests). Eight (57.1%) Alzheimer's disease patients were APOE ɛ4 carriers, with no significance difference between EAD and NEAD groups (*P* = 0.62, Fisher's exact test).

**Table 1:** Demographics of participants analysed

Characteristics	AD	EAD	NEAD	Control
*n*	14	8	6	8
Age, mean (SD), years	59.3 (6.3)	58.9 (7.0)	59.8 (5.8)	71.7 (4.7)
Male, No. (%)	7 (50)	5 (62.5)	2 (33.3)	5 (62.5)
Female, No. (%)	7 (50)	3 (37.5)	4 (66.7)	3 (37.5)
Education level, mean (SD), years	16.7 (3.4)	16.6 (4.1)	17.0 (2.7)	17.0 (1.1)
Handedness, right (%)	12 (85.7)	7 (87.5)	5 (83.3)	7 (87.5)
Race, white, No. (%)	14 (100)	8 (100)	6 (100)	8 (100)
Ethnicity, non-Hispanic, No. (%)	13 (92.8)	7 (87.5)	6 (100)	8 (100)
Acetylcholinesterase use (%)	14 (100)	8 (100)	6 (100)	0 (0)
Memantine use (%)	1 (7.1)	1 (12.5)	0 (0)	0 (0)
MMSE, mean (SD)	24.8 (3.2)	25.2 (2.0)	24.3 (4.6)	29.2 (0.8)
CDR, mean (SD)	0.64 (0.3)	0.62 (0.2)	0.66 (0.4)	0 (0)
CDR-SOB, mean (SD)	3.3 (1.4)	3.6 (0.7)	3.0 (1.9)	0 (0)
APOE ɛ4 carrier, *n* (%)	8 (57.1)	4 (50)	4 (66.6)	N/A

AD, Alzheimer's disease; EAD, epileptic AD; NEAD, non-epileptic AD; CDR, clinical dementia rating; SOB, sum of boxes.

### MEG signal characteristics

**Figure 2:**
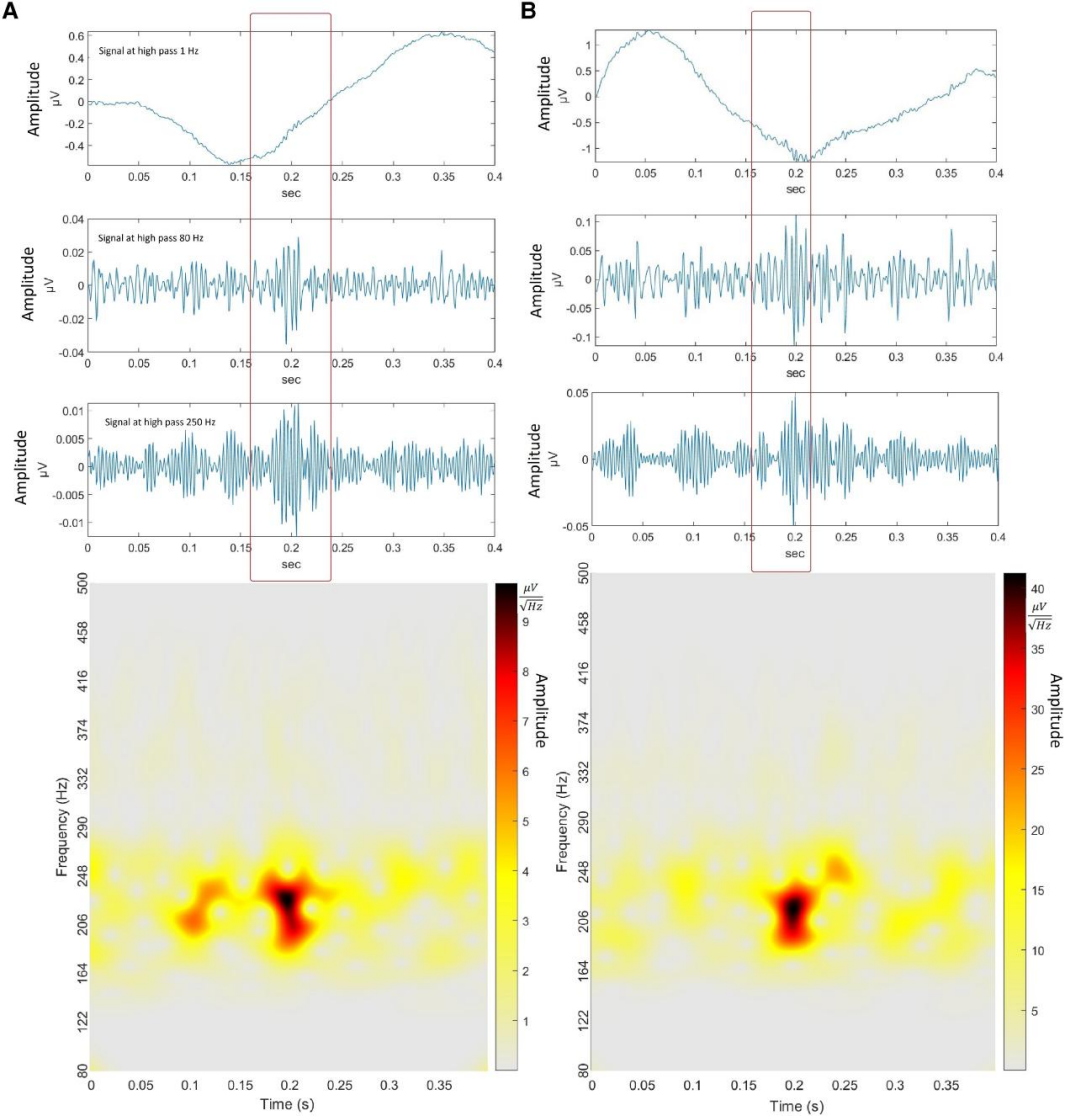
**Typical MEG ripples detected by Delphos detector software.** (**A**) Ripple 200 Hz at right temporal-44 channel and (**B**) ripple 212 Hz at left temporal-51. Top to bottom for each HFO: Signals with high-pass filter at 1, 80 and 250 Hz, and spectrogram of the above signal segment.

**Figure 3:**
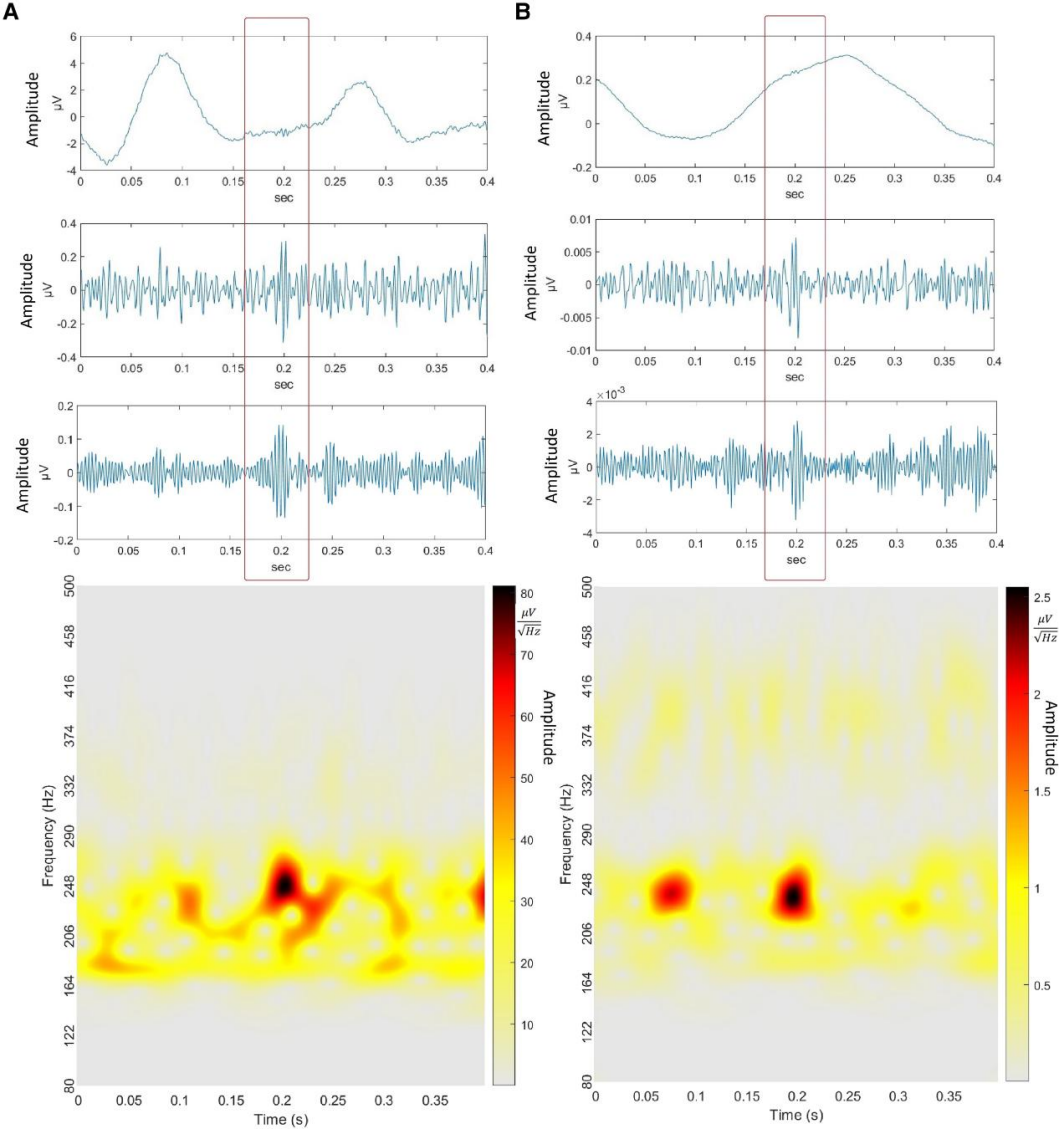
**Typical MEG FR detected by Delphos detector software.** (**A**) FR 267 Hz at right frontal-13. (**B**) FR 252 Hz at left temporal-31. Top to bottom for each HFO: Signals with high-pass filter at 1, 80 and 250 Hz, and spectrogram of the above signal segment.

M/EEG signal had high power at multiples of 60 Hz, indicating the presence of strong electrical noise interference from the power lines or other electronic devices operating at the same frequency. Moreover, while the detected HFO events could be differentiated from the background noise, their prominence was not exceptional, indicating low signal-to-noise ratio. *Delphos detector* identified several oscillations as HFOs. We additionally filtered out the oscillations that had their peak values <3 SD relative to the signal (Supplementary Fig. S2 shows filtered out samples). Figures 2 and 3 illustrates ripples and FR, respectively, from the right temporal region of an EAD participant. Additional example HFOs (R and FR) are shown in Supplementary Fig. S3. Supplementary Fig. S4 shows spatial specificity of a typical HFO. Simultaneous EEG data had low signal-to-noise and was thus excluded from HFO analysis. In our study, four participants were awake, 14 were drowsy (Stage 1 sleep) and 4 were in Stage 2 sleep during the 10-min recordings, with no significant difference between controls, EAD and NEAD (*P* > 0.99, Fisher's exact test). Spearman correlation analysis revealed a range of correlations for rate of ripples from −0.30 to 0.47 across the 11 brain regions, with a median of 0.01 and interquartile range from −0.11 to 0.10. For FR, the range was from −0.48 to 0.39, median of 0.00 and interquartile range from −0.11 to 0.15. This indicated that most correlations were weak, suggesting relative independence among most brain regions.

### Alzheimer's disease participants have more ripples and fast ripples than controls

**Figure 4:**
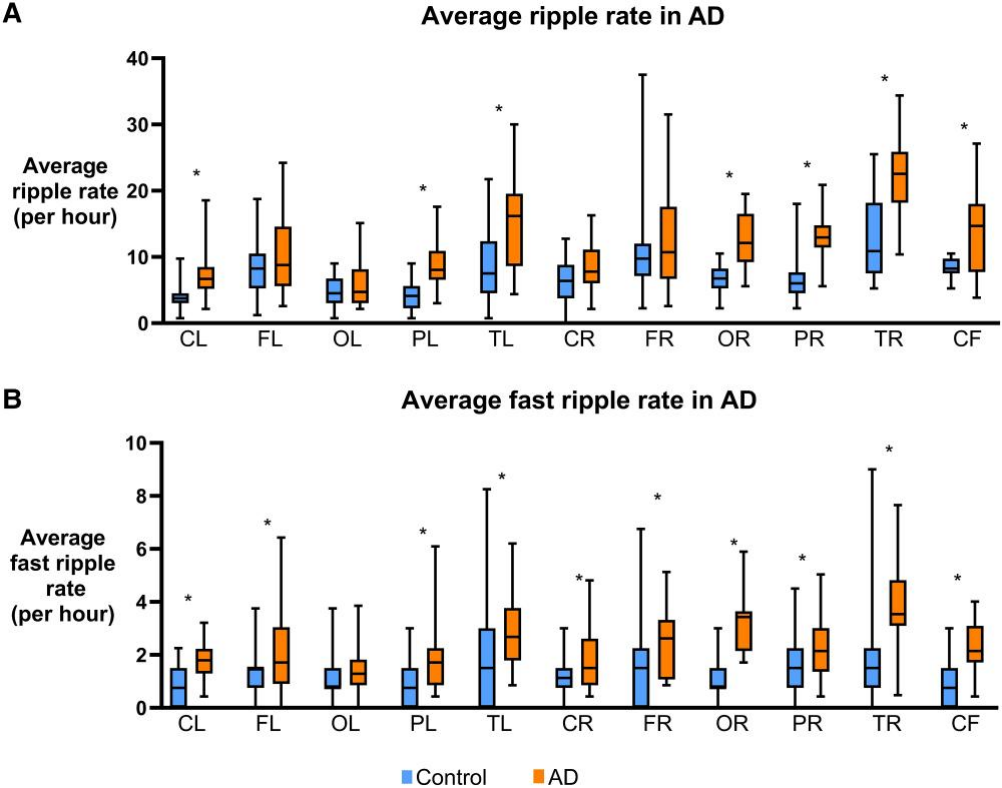
**HFOs in Alzheimer's disease.** (**A**) Average RRs per region detected in MEG for Alzheimer's disease are shown in box plot. Alzheimer's disease had higher RR than controls in central, parietal and temporal regions, and cerebral fissure. (**B**) Average FRR per region detected in MEG for Alzheimer's disease are shown in box plot. Alzheimer's disease had higher FRR than controls in all regions except left occipital. *P* < 0.05, Student's *t*-test or Mann–Whitney test. The average RR or FRR of all participants within a cohort is calculated for each channel, and each regional group of channels is compared between cohorts. Number of channels (**N**) for each region are as follows: CL, central left (n = 24); FL, frontal left (n = 31); OL, occipital left (n = 19); PL, parietal left (n = 22); TL, temporal left (n = 33); CR, central right (n = 24); FR, frontal right (n = 33); OR, occipital right (n = 19); PR, parietal right (n = 22); TR, temporal right (n = 34); CF, cerebral fissure (n = 11).

MEG detected higher average ripple rate (RR) in Alzheimer's disease compared to controls (CN) in the left central (Alzheimer's disease = 7.49, CN = 4.28, *P* < 0.0001, MW), left parietal (Alzheimer's disease = 8.99, CN = 4.33, *P* < 0.0001, ST), left temporal (Alzheimer's disease = 14.78, CN = 8.60, *P* = 0.0001, ST), right occipital (Alzheimer's disease = 12.67, CN = 6.63, *P* < 0.0001, ST), right parietal (Alzheimer's disease = 12.87, CN = 6.61, *P* < 0.0001, ST), right temporal (Alzheimer's disease = 21.95, CN = 13.01, *P* < 0.0001, ST) and cerebral fissure (Alzheimer's disease = 13.30, CN = 8.25, *P* = 0.04, ST) regions (Fig. 4A). Alzheimer's disease participants had higher average FR rate (FRR) than controls in the left central (Alzheimer's disease = 1.79, CN = 0.84, *P* < 0.0001, ST), left frontal (Alzheimer's disease = 2.15, CN = 1.35, *P* = 0.04, MW), left parietal (Alzheimer's disease = 1.81, CN = 0.92, *P* = 0.0059, MW), left temporal (Alzheimer's disease = 2.96, CN = 1.84, *P* = 0.0015, MW), right central (Alzheimer's disease = 1.80, CN = 1.16, *P* = 0.036, ST), right frontal (Alzheimer's disease = 2.49, CN = 1.59, *P* = 0.0021, MW), right occipital (Alzheimer's disease = 3.20, CN = 1.14, *P* < 0.0001, ST), right parietal (Alzheimer's disease = 2.36, CN = 1.47, *P* = 0.01, ST), right temporal (Alzheimer's disease = 3.97, CN = 1.74, *P* < 0.0001, MW) and cerebral fissure (Alzheimer's disease = 2.25, CN = 0.89, *P* = 0.003, ST) regions (Fig. 4B). FR occurred in more lobes than R. Both R and FR were most frequent in right temporal and right occipital regions.

### Both EAD and NEAD participants have higher rates of ripples and fast ripples than controls

**Figure 5:**
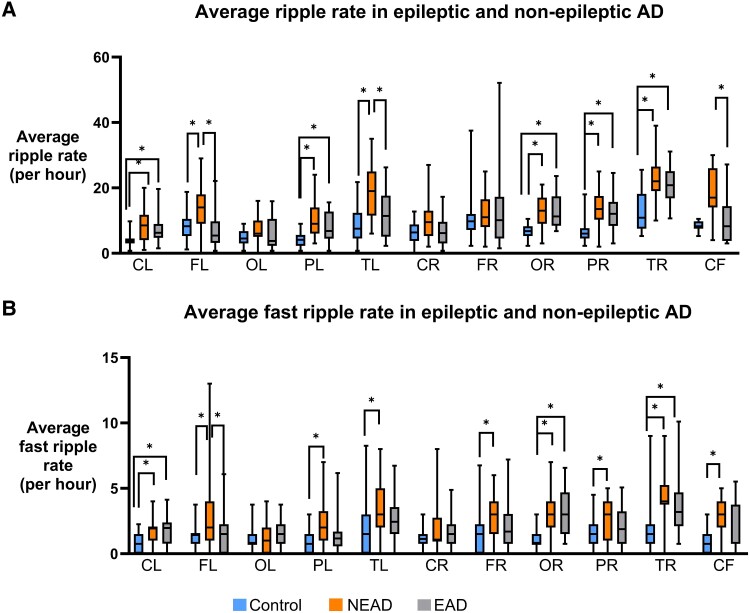
**HFOs in epileptic and non-epileptic Alzheimer's disease.** (**A**) Average RRs per region detected in MEG for EAD and NEAD are shown in box plot. EAD had higher RR than controls in left central, left parietal, right occipital, right parietal and right temporal regions. NEAD had higher RR than controls in all regions except left occipital and right frontal. NEAD had higher RR than EAD in left frontal, left temporal and cerebral fissure. (**B**) Average FRR per region detected in MEG for EAD and NEAD are shown in box plot. EAD had higher FRR than controls in left central, right occipital and right temporal regions. NEAD had higher FRR than controls in all regions except left occipital and right central. NEAD had higher FRR than EAD in left-frontal region. *P* < 0.05, one-way ANOVA/Tukey or Kruskal–Wallis test with Dunn's multiple comparisons test. The average RR or FRR of all participants within a cohort is calculated for each channel, and each regional group of channels is compared between cohorts. Number of channels (**N**) for each region are as follows: CL, central left (n = 24); FL, frontal left (n = 31); OL, occipital left (n = 19); PL, parietal left (n = 22); TL, temporal left (n = 33); CR, central right (n = 24); FR, frontal right (n = 33); OR, occipital right (n = 19); PR, parietal right (n = 22); TR, temporal right (n = 34); CF, cerebral fissure (n = 11).

MEG detected higher RR in EAD than controls in the left central (EAD = 7.07, CN = 4.28, *P* = 0.015, KD), left parietal (EAD = 7.81, CN = 4.33, *P* = 0.016, KD), right occipital (EAD = 12.49, CN = 6.63, *P* = 0.0003, AT), right parietal (EAD = 12.19, CN = 6.61, *P* = 0.0018, KD) and right temporal (EAD = 21.27, CN = 13.01, *P* = 0.0001, AT) regions (Fig. 5A). NEAD had higher RR than controls in the left central (CN = 4.28, NEAD = 8.04, *P* = 0.008, KD), left frontal (CN = 8.38, NEAD = 14.38, *P* = 0.005, KD), left parietal (CN = 4.33, NEAD = 10.54, *P* < 0.0001, KD), left temporal (CN = 8.59, NEAD = 19.00, *P* < 0.0001, AT), right occipital (CN = 6.63, NEAD = 12.89, *P* < 0.0001, AT), right parietal (CN = 6.61, NEAD = 13.77, *P* < 0.0001, KD) and right temporal (CN = 13.01, NEAD = 22.85, *P* < 0.0001, AT) regions. Surprisingly, NEAD had higher RR than EAD in the left frontal (EAD = 7.57, NEAD = 14.38, *P* = 0.0001, KD), left temporal (EAD = 11.61, NEAD = 19.00, *P* < 0.0001, AT) and cerebral fissure (EAD = 9.56, NEAD = 18.27, *P* = 0.037, KD) regions.

EAD had higher FRR than controls in the left central (EAD = 1.82, CN = 0.84, *P* = 0.0035, AT), right occipital (EAD = 3.11, CN = 1.14, *P* = 0.0005, AT) and right temporal (EAD = 3.58, CN = 4.47, *P* = 0.0002, KD) regions (Fig. 5B). NEAD had higher FRR than controls in the left central (CN = 0.84, NEAD = 1.75, *P* = 0. 007, AT), left frontal (CN = 1.35, NEAD = 2.87, *P* = 0.003, KD), left parietal (CN = 0.92, NEAD = 2.31, *P* = 0.013, KD), left temporal (CN = 1.84, NEAD = 3.39, *P* = 0.001, KD), right frontal (CN = 1.59, NEAD = 2.78, *P* = 0.0017, KD), right occipital (CN = 1.14, NEAD = 3.31, *P* < 0.0001, AT), right parietal (CN = 1.46, NEAD = 2.63, *P* = 0.022, AT), right temporal (CN = 1.74, NEAD = 4.47, *P* < 0.0001, KD) and cerebral fissure (CN = 0.88, NEAD = 2.72, *P* = 0.015) regions. NEAD had higher FRR than EAD in the left-frontal (EAD = 1.64, NEAD = 2.87, *P* = 0.025) region.

### LEV decreased ripples in EAD, but increased ripples and fast ripples in NEAD

**Figure 6:**
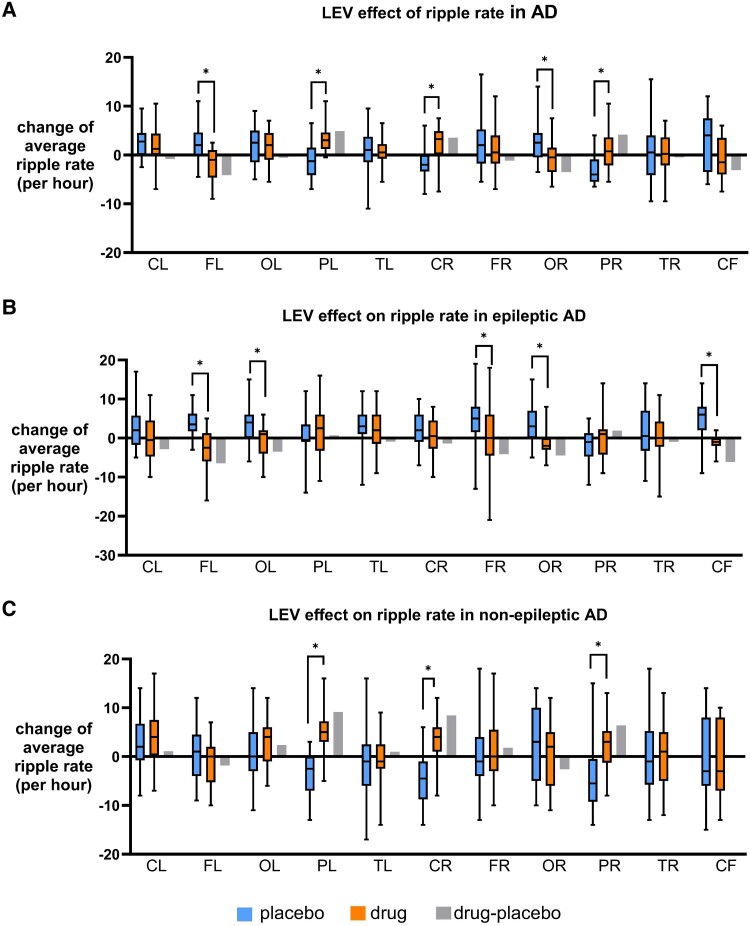
**Effect of LEV on ripples.** (**A**) Average RRs per region detected in MEG for Alzheimer's disease before and after LEV treatment are shown in box plot. LEV increased RR in Alzheimer's disease in bilateral parietal and right central but decreased RR in left-frontal and right-occipital regions. (**B**) Average RRs per region detected in MEG for EAD before and after LEV treatment are shown in box plot. In EAD, LEV decreased RR in bilateral-frontal and occipital regions and cerebral fissure. (**C**) Average RRs per region detected in MEG for NEAD before and after LEV treatment are shown in box plot. In NEAD, LEV increased RR in bilateral parietal and right central regions. *P* < 0.05, unpaired *t*-test or Mann–Whitney test. The average RR or FRR of all participants within a cohort is calculated for each channel, and each regional group of channels is compared between cohorts. Number of channels (**N**) for each region are as follows: CL, central left (n = 24); FL, frontal left (n = 31); OL, occipital left (n = 19); PL, parietal left (n = 22); TL, temporal left (n = 33); CR, central right (n = 24); FR, frontal right (n = 33); OR, occipital right (n = 19); PR, parietal right (n = 22); TR, temporal right (n = 34); CF, cerebral fissure (n = 11).

In Alzheimer's disease, LEV decreased R in the left frontal (*P* < 0.0001) and right occipital (*P* = 0.017) regions and increased R in the left parietal (*P* < 0.0001), right parietal (*P* = 0.0003) and right central (*P* = 0.0007) regions (ST) (Fig. 6A). In EAD, LEV decreased R in the left frontal (*P* < 0.0001, ST), right frontal (*P* = 0.013, ST), left occipital (*P* = 0.036, ST), right occipital (*P* = 0.002, MW) and cerebral fissure (*P* = 0.009, ST) regions (Fig. 6B). However, in NEAD, LEV increased R in the left parietal (*P* < 0.0001, ST), right parietal (*P* = 0.0002, MW) and right central (*P* < 0.0001, ST) regions (Fig. 6C).

**Figure 7:**
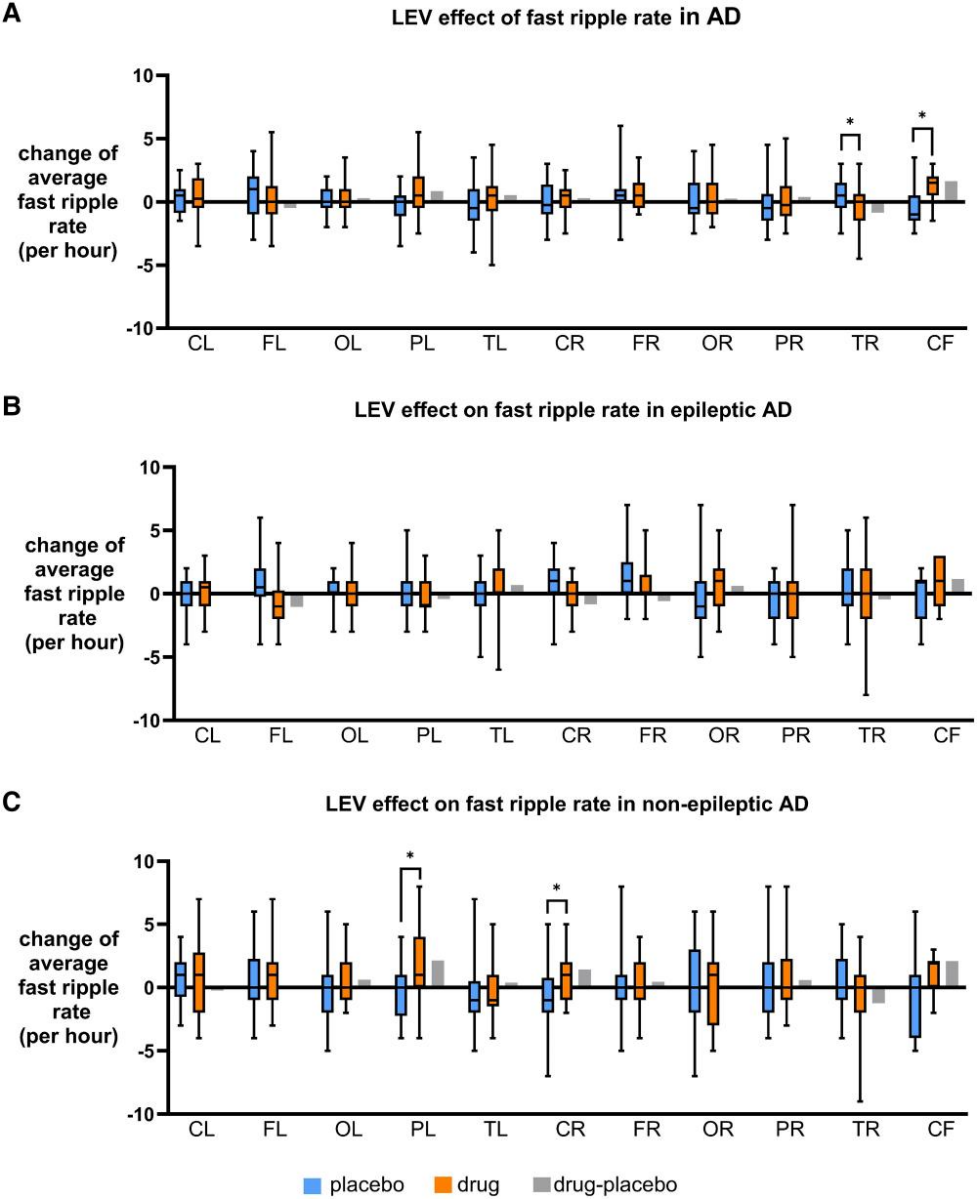
**Effect of LEV on FR.** (**A**) Average FRRs per region detected in MEG for Alzheimer's disease before and after LEV treatment are shown in box plot. LEV increased RR in Alzheimer's disease in bilateral parietal and right central but decreased RR in left-frontal and right-occipital regions. (**B**) Average FRRs per region detected in MEG for EAD before and after LEV treatment are shown in box plot. In EAD, LEV decreased RR in bilateral-frontal and occipital regions and cerebral fissure. (**C**) Average FRRs per region detected in MEG for NEAD before and after LEV treatment are shown in box plot. In NEAD, LEV increased RR in bilateral parietal and right central regions. *P* < 0.05, unpaired *t*-test or Mann–Whitney test. The average RR or FRR of all participants within a cohort is calculated for each channel, and each regional group of channels is compared between cohorts. Number of channels (**N**) for each region are as follows: CL, central left (n = 24); FL, frontal left (n = 31); OL, occipital left (n = 19); PL, parietal left (n = 22); TL, temporal left (n = 33); CR, central right (n = 24); FR, frontal right (n = 33); OR, occipital right (n = 19); PR, parietal right (n = 22); TR, temporal right (n = 34); CF, cerebral fissure (n = 11).

In Alzheimer's disease, LEV decreased FR in right temporal region (*P* = 0.03, ST) but increased them in cerebral fissure (*P* = 0.02, MW) (Fig. 7A). For FR, in EAD, LEV did not show any effect (Fig. 7B). In NEAD, LEV increased FR in the left parietal (*P* = 0.006) and right central (*P* = 0.035) regions (ST) (Fig. 7C); both regions showed increase in R too.

### Asymmetry index of HFOs

**Figure 8:**
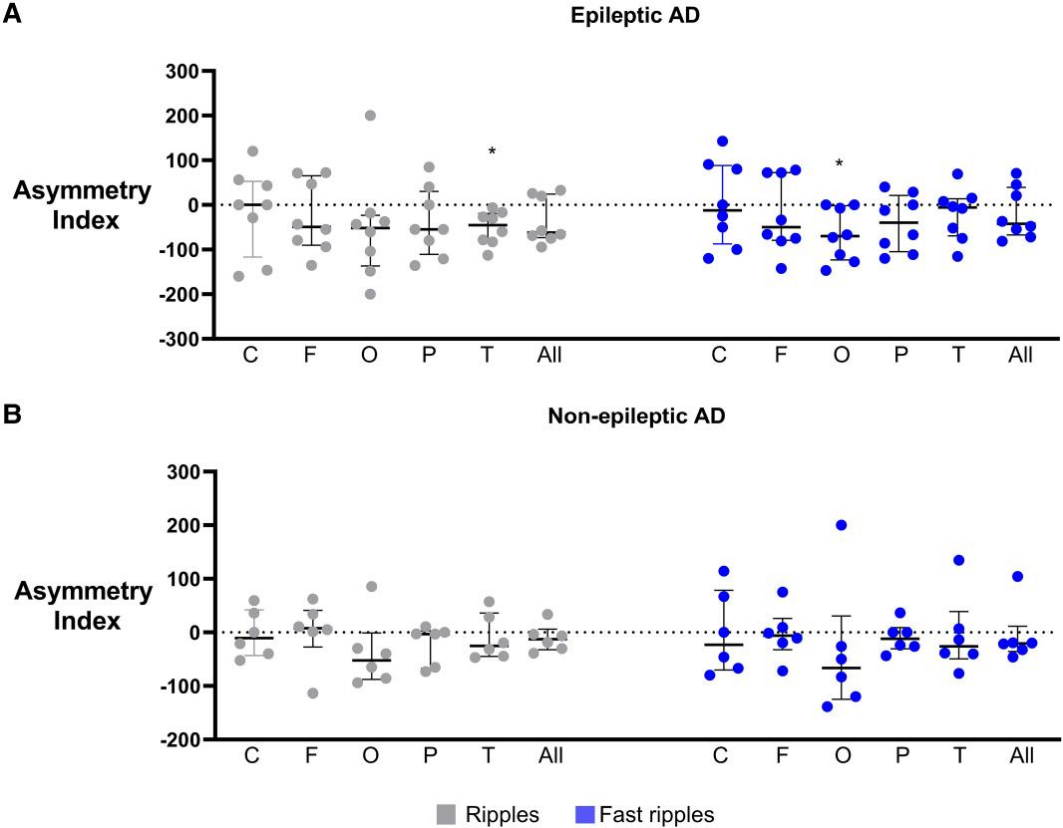
**AI of EAD and NEAD.** AI of ripples and FR in (**A**) EAD showed significant asymmetry in occipital and temporal regions, with higher HFOs in the right hemisphere. AI of (**B**) NEAD shows that there is no asymmetry in any region. N = 8 and 6 for EAD and NEAD, respectively. *P* < 0.05, 2-tailed 1-sample *t*-test versus zero. C, central; F, frontal; O, occipital; P, parietal; T, temporal; All, all regions combined.

EEG showed epileptic activity in the bilateral frontal region (n = 1) and predominantly left temporal region (n = 5) for EAD participants, where MEG showed bilateral temporal (n = 1) and left temporal (n = 1) epileptic activity. A patient in the EAD group had a recent history of seizures but no epileptic activity during M/EEG recording. We compared AI of EAD against the AI of NEAD. There was no significant difference between AIs of EAD and NEAD for both R (*P* value for regions is 0.79 for central, 0.50 for frontal, 0.81 for occipital, 0.61 for parietal, 0.07 for temporal and 0.30 for whole hemisphere) and FR (*P* value for regions is 0.92 for central, 0.63 for frontal, 0.55 for occipital, 0.27 for parietal, 0.67 for temporal and 0.66 for whole hemisphere; ST). We also compared AI of EAD and NEAD against zero for assessing absolute asymmetry. We found higher R in the right hemisphere for EAD in the temporal region (*P* = 0.005; 1-sample *t*-test). There was no asymmetry for R in NEAD. We found higher FR in the right hemisphere for EAD in the occipital region (*P* = 0.015; 1-sample *t*-test). There was no asymmetry for FR in NEAD (Fig. 8).

## Discussion

HFOs have so far not been observed in MEG recordings that are not associated with epilepsy until now. In this study, we showed that Alzheimer's disease exhibits more R and FR in several brain regions. This finding is in line with the current view that neuronal hyperactivity is associated with Alzheimer's disease.^3^ We also noted that FR occur in more lobes than R in Alzheimer's disease, which is in line with the view that FR are more often pathological and expected to be more pronounced in disease conditions.^29^ Several regions showed more HFOs in Alzheimer's disease; however, not all lobes were affected to the same degree. The right temporal and right occipital regions showed greater difference than controls in both R and FR. This could be due to the pathological process, including tauopathy, affecting the region in the early stages and spreading to rest of the brain,^49^ implying that tauopathy could influence the prevalence or intensity of HFOs in affected brain regions. Our results also confirm the findings in mouse models that showed Alzheimer's disease and epilepsy models had higher HFOs than controls.^50^ Additionally, the dentate gyrus showed robust HFO activity in mouse models, which is similar to the temporal lobe having higher HFOs in our study. NEAD had higher HFO rates than EAD in a few regions. We hypothesize that compensatory alterations in metabolic activity and molecular expressions, for example, relatively higher levels of neuropeptide Y,^51,52^ can reduce wider network hypersynchrony in these cases. We found significant asymmetry of HFOs in EAD, but not in NEAD. EAD had higher HFO rates, for both R (in occipital and temporal regions) and FR (in occipital region), in the right hemisphere. This is in line with a previous study in Alzheimer's disease,^46^ which showed that epileptiform discharges detected by MEG were more on the right hemisphere (temporal lobe); whereas EEG detected epileptiform discharges were predominant on the left side. HFOs occur in both awake and sleep states: REM and Non-REM, although their rates are higher in non-REM sleep, followed by awake and then REM sleep.^53^ R are more affected by sleep/wake state than FR. In our study, there was no difference in participants’ awake/sleep states. FR were present in our control group, who were on average 12 years older than the disease group. While often associated with pathology, FR might also have physiological origins. These findings could potentially represent early signs of hyperexcitability in the aging brain, which might progress to seizure activity. This observation underscores the complexity of distinguishing age-related changes from early pathological processes, suggesting a need for further investigation. We also observed that HFO rates are uncorrelated across brain regions, leading us to treat each region as independent in our analysis. Future studies should examine interregional dependencies before investigating intraregional effects.

We know that LEV can reduce HFOs in the rat model of temporal lobe epilepsy.^54^ In this study, we showed that LEV decreased R HFOs in human epileptic Alzheimer's disease. At our given dose, LEV did not reduce HFOs in all the brain regions that were affected at baseline. It needs to be tested if a different dose can extend LEV's affect across all brain regions in EAD. Surprisingly, LEV differently affected NEAD where it increased the HFOs for both R and FR. This implies that LEV is influencing at least two different mechanisms underlying hyperactivity. This aligns with our previous findings from this same study,^4^ in which, Alzheimer's disease with epileptic activity and Alzheimer's disease without epileptic activity were differently affected by LEV in their measures of cognitive function. For Alzheimer's disease with epileptic activity, compared to placebo, LEV increased performance in the Stroop interference naming test (SINT) and increased learning rates in the virtual route learning test (VRLT), whereas Alzheimer's disease without epileptic activity did not received any benefit from LEV. Compared to Alzheimer's disease without epileptic activity, Alzheimer's disease with epileptic activity had better SINT performance and higher accuracy in VRLT while taking LEV. One possibility for the differential effects of LEV on HFOs in NEAD and EAD is differences in brain SV2A levels between NEAD and EAD. Brain SV2A levels are decreased in autopsy tissue of epilepsy patients,^55^ and could be more diminished in EAD than in NEAD. Accordingly, LEV's impact could be higher if SV2A levels are lower to start with in EAD. Moreover, the epileptic sub-type of Alzheimer's disease exhibits distinct neurophysiological characteristics particularly in the expression and function of various ion channels and neurotransmitter systems.^56^ These alterations create a unique neural environment that differentiates this sub-type from non-epileptic Alzheimer's disease. Such changes can emerge early in the disease process and can be exacerbated by seizure activity. More insight is needed on the dynamics of synaptic inhibitory processes between the EAD, NEAD and non-AD epileptic systems. Our MEG study captured HFOs that likely represent a mixture of cortical and hippocampal ripples, as MEG sensors aggregate activity from both deep structures and neocortical regions. While our findings demonstrate regional variations in HFO patterns, future studies combining MEG with detailed clinical phenotyping and neuroimaging could help understand whether these patterns differ between Alzheimer's disease variants (e.g. predominantly medial temporal versus neocortical). Additionally, combining MEG with techniques offering higher spatial resolution, such as simultaneous intracranial EEG, could help distinguish the sources and characteristics of cortical versus hippocampal ripples.

### Limitations

The age of control participants was higher than Alzheimer's disease. However, considering pathological nature of HFOs, one could expect their direct relationship with age, but controls had less HFOs. The MEG analysis was limited by noise, and a better signal-to-noise ratio would be more sensitive to detect HFOs by morphology. An HFO study will also benefit from higher sample sizes in more diverse populations. Also, 10-minute M/EEG exams cannot capture all vigilance states in which HFOs can occur. In future studies, longer M/EEG recordings, with high sampling rates, would help to identify HFOs better.

## Conclusion and future directions

In this study, we found that Alzheimer's disease exhibited a higher rate of HFOs, with intriguing differences between NEAD and EAD. LEV decreased HFOs in EAD but increased them in NEAD, suggesting that HFOs associated with epileptic activity and asymmetry in Alzheimer's disease are more pathological. Additionally, LEV treatment could increase hyperexcitability in parietal/central regions of non-epileptic Alzheimer's disease cases. While HFOs can serve as a biomarker of hyperexcitability in Alzheimer's disease, their role in Alzheimer's disease pathology and progression requires further investigation. Improved methods for objectively assessing HFOs and handling technique-specific noise, especially for scalp EEG and MEG are needed. Overall, we present strong evidence that HFOs are one of the neurophysiological biomarkers of Alzheimer's disease, and they can be used to study other HFO-correlated molecular and neurophysiological variables in this disease.

## Supplementary Material

fcaf041_Supplementary_Data

## Data Availability

The data that support the findings of this study are available from the corresponding author, upon reasonable request. The software script is available at https://github.com/Vossel-Lab/HFO-AD-Lev.git
