# Supplementary material for: High-frequency oscillations in epileptic and non-epileptic Alzheimer's disease patients and the differential effect of levetiracetam on the oscillations
Source: Brain Commun. 2025 Feb 13;7(1):fcaf041. doi: 10.1093/braincomms/fcaf041 (PMC11822293; doi:10.1093/braincomms/fcaf041)
Supplement: fcaf041_Supplementary_Data [file fcaf041_supplementary_data.pdf]

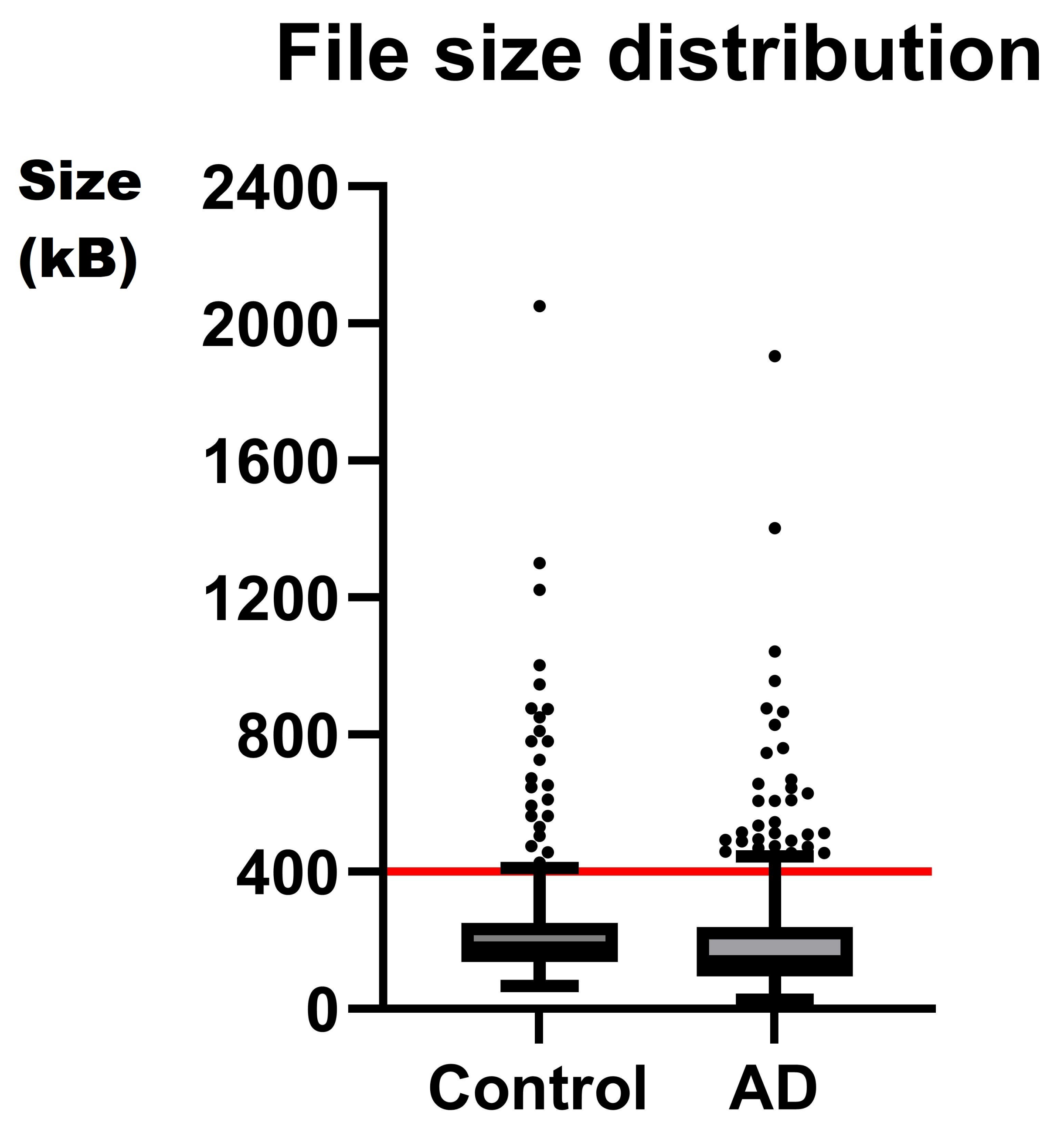

Supplementary Fig. 1. Distribution of sizes of files with the list of HFO candidates. The bars of box plot represent outliers using the Tukey method. The red line at 400 kB was the cutoff size that was applied, where files larger than that were excluded. N = 199 and 474 for control and Alzheimer's disease, respectively.

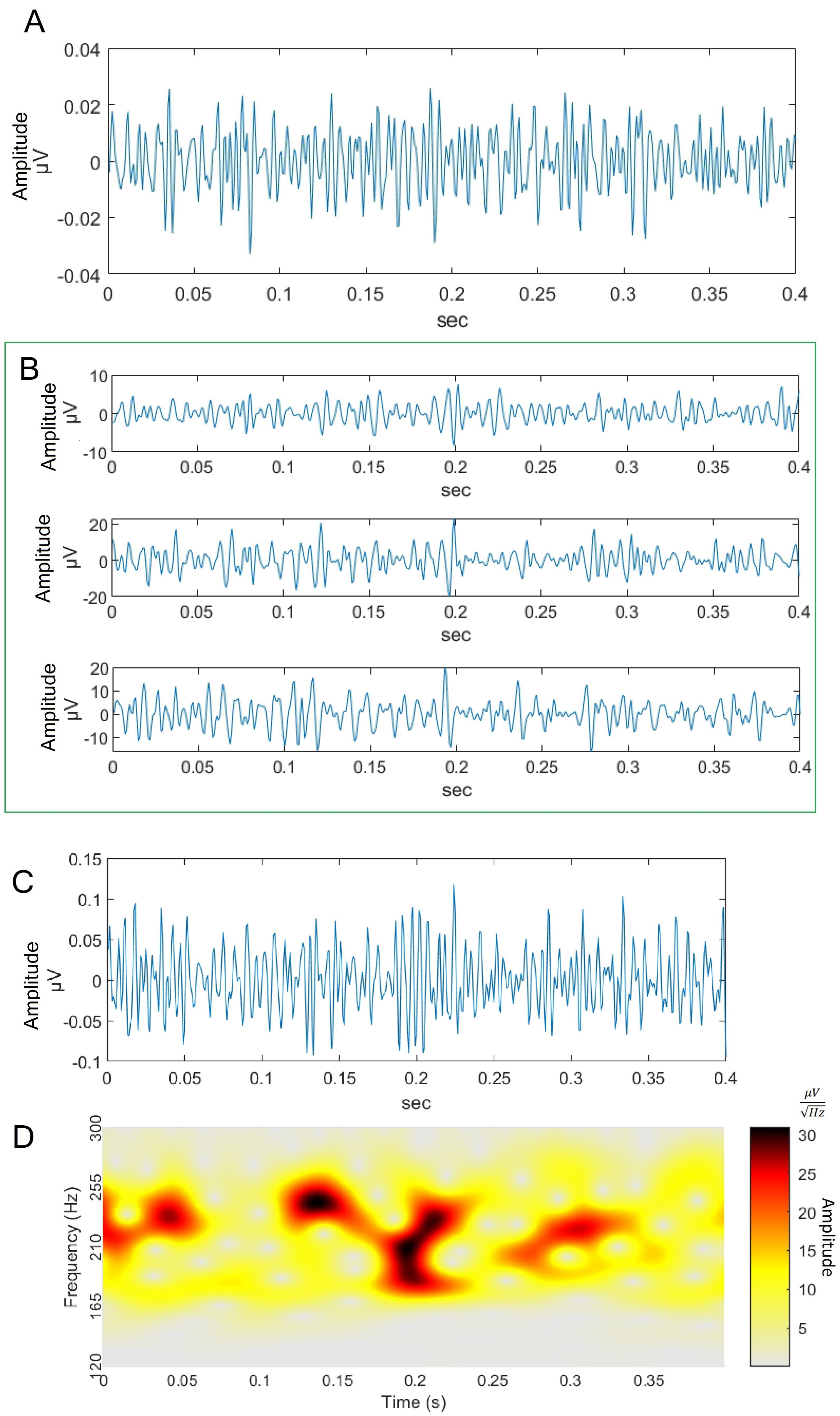

Supplementary Fig. 2. Sample signals that were filtered out at different stages. (**A**) Not detected by *Delphos detector* script; (**B**) for having more than five candidates across different channels (three random channels shown: left frontal-45, right temporal-22, right parietal-56 (top to bottom)); (**C**) having high frequency component with amplitude less than three SD and (**D**) its spectrogram. µV = microvolts.

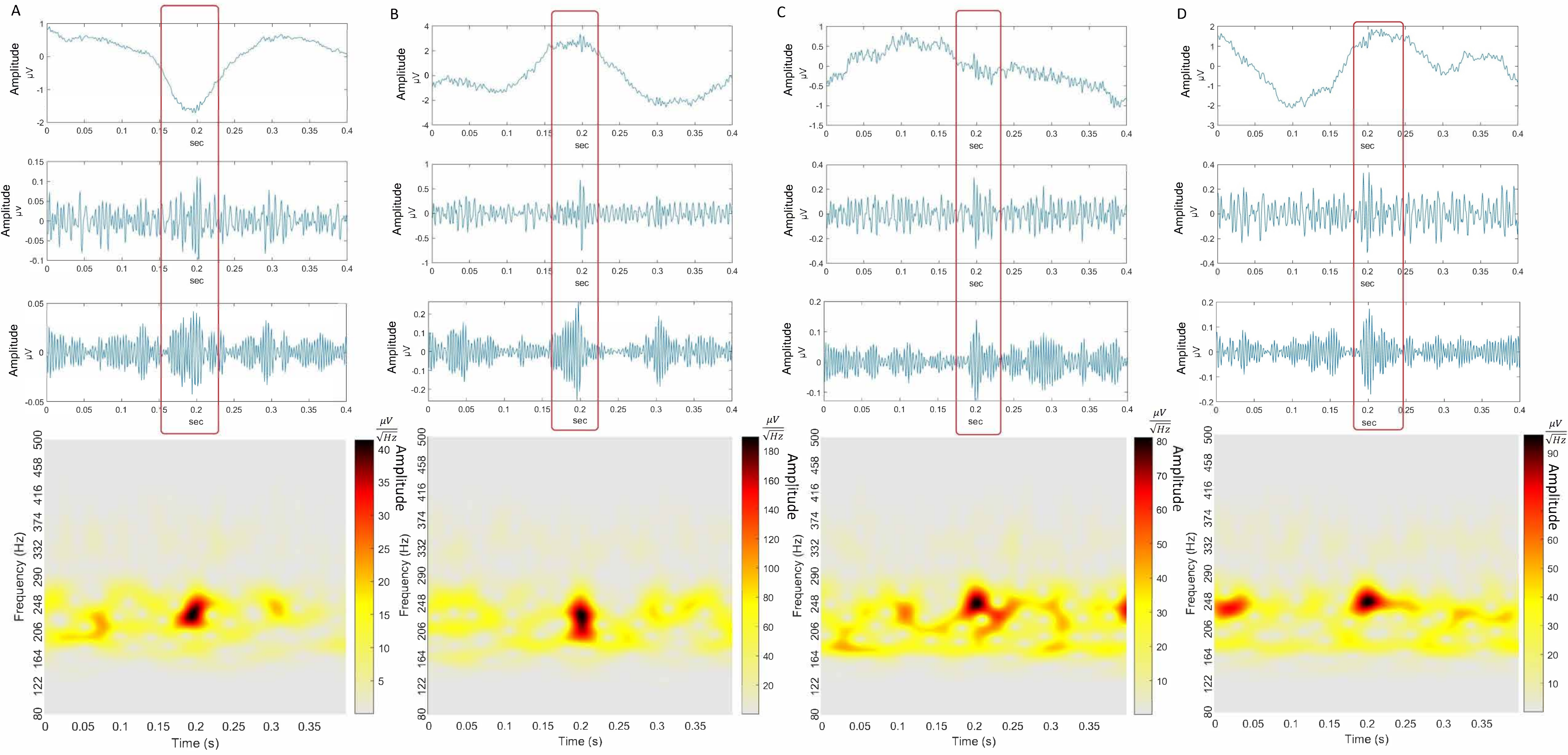

Supplementary Fig. 3. Sample HFOs. [top to bottom] Signals with high-pass filter at 1 Hz, at 80 Hz, at 250 Hz, and a spectrogram for ripple 238 Hz at right temporal-44 of epileptic AD (**A**), ripple 224 Hz at left temporal-52 of non-epileptic AD (**B**), fast ripple 252 Hz at right parietal-45 of epileptic AD (**C**) and fast ripple 267 Hz at left central-23 of non-epileptic AD (**D**). HFOs are shown in red box. µV = microvolts.

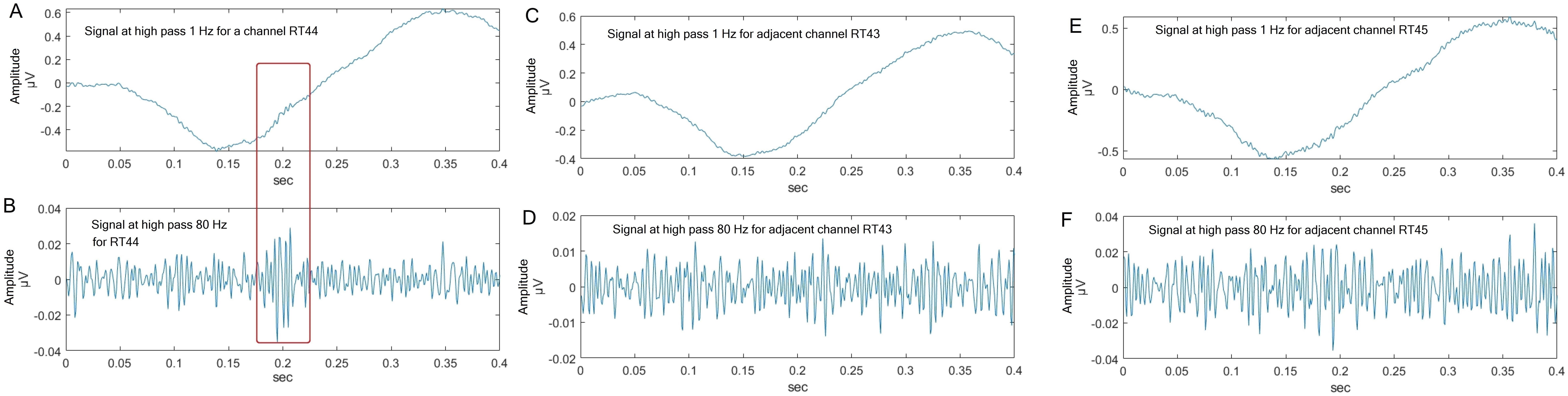

Supplementary Fig. 4. Spatial specificity of a typical HFO. (**A**, **B**) Ripple HFO (200 Hz) at RT44 channel (in red box). Signals at the same moment of time from some adjacent channels, with no HFOs, are also shown. (**A**) HFO signal with high-pass filter at 1 Hz and (**B**) high-pass filter at 80 Hz. Signals of adjacent channels RT43 (**C**, **D**) and RT45 (**F**, **G**) with high-pass filter at 1 Hz (**C, E**) and at 80 Hz (**D, F**). μV = microvolts; RT = right temporal region.
